# A Single Microorganism Epitope Attenuates the Development of Murine Autoimmune Arthritis: Regulation of Dendritic Cells *via* the Mannose Receptor

**DOI:** 10.3389/fimmu.2018.01528

**Published:** 2018-07-02

**Authors:** Fan Yang, Xuemei Fan, He Huang, Qiujie Dang, Hongwei Lei, Yang Li

**Affiliations:** Department of Rheumatology and Immunology, The Second Affiliated Hospital of Harbin Medical University, Harbin, China

**Keywords:** arthritis, *Leishmania majo*r, dendritic cells, Jagged1, mannose receptor

## Abstract

A single epitope of *Leishmania* analog of the receptors for activated C kinase (LACK) from *Leishmania major*, the polypeptide LACK_156–173_, is recognized by Vβ4^+^/Vα8^+^ T cells, and activate these cells that drives the subsequent T helper (Th)2 response. This study was undertaken to investigate the therapeutic potential of the LACK_156–173_ epitope in murine autoimmune arthritis models. To explore the influence of the LACK_156–173_ epitope on murine collagen antibody-induced arthritis, as well as its immunological mechanism, we vaccinated or treated mice with a LACK_156–173_ epitope expression plasmid or polypeptide. The effect of LACK_156–173_ epitope was then evaluated by clinical scores, histopathology, and quantitative real-time polymerase chain reaction (qRT-PCR) analysis. Using flow cytometry, we measured the subsets and maturity of CD11c^+^ dendritic cells (DCs), as well as T cell polarization, in co-culture experiments. We also measured cytokine gene expression and production. The murine macrophage-like cell line RAW264.7 was used to identify the receptor for the epitope. Vaccination or treatment of the mice with the LACK_156–173_ epitope expression plasmid or polypeptide ameliorated the severity of arthritis. qRT-PCR analysis revealed that the LACK_156–173_ epitope improved the balance of effector T cells in synovial tissue compared to that in untreated arthritis controls. Toll-like receptor (TLR) 4 expression was diminished by LACK_156–173_. The epitope also influenced T cell polarization by regulating the differentiation, maturation, and functions of CD11c^+^ DCs and upregulating Jagged1 ligand expression. Blocking the mannose receptor (MR) significantly attenuated LACK_156–173_ epitope-induced macrophage activation. Our data indicate that vaccination or treatment with a single microorganism epitope, LACK_156–173_, is a highly efficient therapy for murine autoimmune arthritis. The therapeutic effects are mediated by the regulation of the differentiation, maturation, and functions of DCs *via* MR, resulting in the upregulation of Jagged1 expression and Th2 cell polarization. Our results demonstrate the therapeutic potential of the LACK_156–173_ epitope in rheumatoid arthritis.

## Introduction

Rheumatoid arthritis (RA) is a chronic autoimmune disease characterized by synovitis and bone destruction; it leads to substantial disability. RA affects approximately 1% of the global population. Although the exact pathogenesis of RA remains unclear, T cell-mediated adaptive immunity is deeply involved in the initiation and progression of RA (1). T helper (Th) 1 and Th17 subsets of CD4^+^ T cells, and the pro-inflammatory cytokines they produce, play critical roles in the pathogenesis of RA (2). However, Th2 immune responses and regulatory T cells have protective effects in RA (3, 4). Therefore, the regulation of T cell responses is an important new treatment strategy in RA.

The “hygiene hypothesis,” which proposes that improvements in public health in the developed world may be causing a rise in the prevalence of autoimmune diseases, has been recognized for two decades (5). This theory has been strengthened by solid epidemiological, experimental, and clinical data, which have paved the way for the development of future therapies. Infections may protect from autoimmune diseases. This paradoxical effect has been demonstrated for a number of bacteria, viruses, and parasites in a variety of spontaneous or experimentally induced animal models of autoimmune diseases (e.g., in animal models of experimental autoimmune encephalomyelitis and inflammatory bowel disease, and in non-obese diabetic mice) (6–11). Some microorganisms skew the immune system toward Th2 responses, and/or the production of interleukin (IL)-4, IL-13, IL-5, and IL-10. Both Th1 and Th17 inflammatory responses and cytokine (TNF-α, IFN-γ, IL-6, IL-12, and IL-17) secretion are inhibited by parasite infections. Some microorganisms also promote a regulatory phenotype of B cells, dendritic cells (DCs), and macrophages. Tolerogenic DCs and regulatory M2 macrophages could all contribute to switching from a Th1/Th17 response to a Th2/Treg profile (12, 13). Therefore, it is a growing and exciting strategy regarding the key role played by microorganisms in autoimmunity.

LACK, the *Leishmania* analog of the receptors for activated C kinase, is an important antigen from *Leishmania major* (14). A single epitope of the LACK protein, polypeptide LACK_156–173_, can be recognized by Vβ4^+^/Vα8^+^ T cells, thereby driving the cells into a Th2 response in susceptible BALB/c mice. The aberrant development of Th2 cells instead of Th1 cells in response to infection renders BALB/c mice unable to control parasite dissemination (15–17). However, the Th2-polarizing epitope may have therapeutic potential in autoimmune diseases. In the present study, we analyzed the effects of the LACK_156–173_ epitope in murine autoimmune arthritis models, and the influence on DC subsets and T cells (18, 19), to determine the therapeutic potential for autoimmune arthritis.

## Materials and Methods

### Mice

We used female BALB/c mice (6–8 weeks old and 16–24 weeks old) and male DBA/1 mice (8−10 weeks old). The mice were purchased from the Experimental Animal Center of The Second Affiliated Hospital of Harbin Medical University. All mice were housed under a 12-h light/12-h dark cycle in a temperature- and humidity-controlled room.

### Arthritis Induction and Assessment

#### Induction of Murine Arthritis Models

Collagen antibody-induced arthritis (CAIA) was induced in female BALB/c mice (6–8 weeks old) by tail vein injection of 1.5 mg anti-type II collagen 5-clone monoclonal antibody (mAb) cocktail (Chondrex, Redmond, WA, USA) on day 0. On day 3, mice received an intraperitoneal (i.p.) injection of 25 µg lipopolysaccharide (LPS; Chondrex, Redmond, WA, USA). The mice were scored daily after the injection of the mAb until day 14.

Proteoglycan (PG)-induced arthritis (PGIA) was induced in female BALB/c mice (16–24 weeks old) *via* 3 i.p. injections of 100 µg bovine PG (Sigma-Aldrich, St. Louis, MO, USA) emulsified in 1 mg dimethyldioctadecylammonium bromide (DDA) (Sigma-Aldrich, St. Louis, MO, USA) adjuvant at intervals of 21 days. After the third injection, the mice were scored daily and evaluated until 70 days after the first injection.

Collagen-induced arthritis (CIA) was induced in DBA/1 mice. Briefly, bovine collagen II (CII) solution (2 mg/ml in 0.05 M acetic acid) (Chondrex, Redmond, WA, USA) was emulsified with an equal volume of Freund’s complete adjuvant (Chondrex, Redmond, WA, USA). On day 0, we subcutaneously (s.c.) injected 0.1 ml of the emulsion at the base of the mouse tail. On day 21, a booster injection (0.1 ml CII emulsified with Freund’s incomplete adjuvant) was administered near the primary injection site. After the booster injection, the mice were scored daily until 50 days after the beginning of arthritis induction.

#### LACK_156–173_ Epitope Vaccination and Treatment

The LACK_156–173_ peptide (ICFSPSLEHPIVVSGSWD, rLACK) and the LACK-K164 mutant peptide (ICFSPSLEKPIVVSGSWD, LACK-K), with the designated alteration of H at position 164, were synthesized by Geni Biotechnology Company (Shanghai, China). The purity of the peptides was greater than 95%.

The recombinant LACK_156–173_ polypeptide expression plasmid pLACK was synthesized by Shanghai CPG Biotech Company and the target gene was sub-cloned into the eukaryotic expression vector pcDNA3.1(+). The plasmid was amplified in TOP10 *Escherichia coli* competent cells. The plasmids were purified in large quantities using EndoFree^®^ Plasmid Maxi Kit (QIAGEN, Hilden, Germany). The presence of the target oligonucleotides was confirmed by DNA sequencing.

Collagen antibody-induced arthritis mice were vaccinated 7 days before mAb injection, with either plasmid or peptide. The mice in the treatment groups were injected with plasmid or peptide on day 4 after mAb injection. PGIA mice were treated with peptide after the third injection of PG with 7-day interval. CIA mice were treated with peptide after the booster injection with 7-day interval.

The plasmid-vaccinated or -treated mice were injected with 50 µl bupivacaine (0.25%) in each quadriceps. Then, 24 h later, mice were injected intramuscularly with 50 µg pLACK (1 µg/µl) or pcDNA3.1(+) per leg in the same place (20). The peptide-vaccinated or -treated mice were injected s.c. in the foot pad with 25 µg rLACK (1 µg/µl) or LACK-K on each side. The mice injected with PBS were used as the control group.

#### Clinical and Histological Assessment of Arthritis

The severity of arthritis in all four paws was graded independently on a 0–4 scale for each paw: 0 = no swelling; 1 = mild erythema or swelling of the wrist or ankle, or erythema and swelling of any severity for one digit; 2 = more than three inflamed digits, or moderate erythema and swelling of the ankle or wrist; 3 = severe erythema and swelling of the wrist or ankle; and 4 = complete erythema and swelling of the wrist and ankle, including all digits.

Limbs of the mice from each group were gotten on the day of sacrifice, which fixed in 10% neutral formalin, decalcified in 15% EDTA solution, and embedded in paraffin. Serial sections were prepared and stained with hematoxylin and eosin.

### Real-Time Polymerase Chain Reaction

The patella and adjacent synovial tissue were dissected from euthanized animals, and ground in liquid nitrogen. Total RNA was isolated using TRIzol™ reagent. Complementary DNA was synthesized from total RNA using AccuPower^®^ RocketScript™ RT Premix (Bioneer, Daejeon, Korea). For the detection of gene transcripts, quantitative real-time polymerase chain reaction (qRT-PCR) was carried out with the AccuPower^®^ 2X SYBR^®^ Green I qPCR Master Mix (Bioneer, Daejeon, Korea). The mRNA expression was normalized to that of housekeeping genes (Actb and Gapdh) by the threshold cycle (ΔΔCT) method.

### Serum Analyses

Anti-LACK_156–173_ epitope antibodies in the serum were quantified by enzyme-linked immunosorbent assay (ELISA). In brief, 96-well plates were coated overnight at 4°C with 10 µg rLACK/ml in PBS. We washed the plates with Tris-buffered saline (pH 7.4) containing 0.1% TWEEN^®^ 20. The sera were tested in duplicate. The amounts of bound IgG antibodies were determined after incubation with polyclonal goat anti-mouse IgG mAb conjugated to horseradish peroxidase. TMB (Sigma-Aldrich, St. Louis, MO, USA) was used as the chromogenic substrate and absorbance was measured at 450 nm with a Multiskan™ Spectrum Microplate reader (Thermo Scientific, Carlsbad, CA, USA).

### Analysis of the Phenotypes of DCs and the *In Vitro* Polarization of T Cells From CAIA Mice

Single-lymphocyte suspensions from the spleens of CAIA mice were generated by mechanical disruption and red blood cell lysis 7 days after mAb injection. LACK_156–173_ epitope-derived CD11c^+^ DCs were purified from the splenocytes of CAIA mice by magnetic cell sorting (Miltenyi, Teterow, Germany). The expression of the surface molecules on the DCs was measured by flow cytometry.

For the polarization of T cells *in vitro*, LACK_156–173_ epitope-derived CD11c^+^ DCs were co-cultured with T cells at a DC:T cell ratio of 1:10. The T cells were isolated by passing splenocytes, from BALB/c mice that had been i.p. injected with 100 µg OVA_323–339_ (Sigma-Aldrich, St. Louis, MO, USA) 7 days prior, through nylon wool fiber in a syringe. The cells were cultured in 24-well culture plates (2 × 10^6^/ml) in RPMI-1640 supplemented with 10% fetal bovine serum (FBS) and OVA_323–339_ (15 µg/ml) for 72 h. The mRNA expression of cytokines and the differentiation of T cells were measured by qRT-PCR and flow cytometry, respectively. We also tested the concentrations of cytokines in the culture supernatants.

### Analysis of T Cell Polarization Induced by Targeting Jagged1 Expression in DCs

Bone marrow-derived dendritic cells (BMDCs) generation: bone marrow cells from the tibiae and femurs of BALB/c mice were cultured with granulocyte–macrophage colony-stimulating factor (20 ng/ml) containing RPMI-1640 medium, with fresh medium added on days 3 and 6. After 9 days, non-adherent cells were collected and stimulated with LPS (1 µg/ml) for 48 h to induce maturation.

Three siRNAs targeting different regions of Jagged1 mRNA were designed and purchased (RiboBio, Guangzhou, China) together with a scrambled siRNA to serve as a negative control. Harvested BMDCs were transfected with the individual siRNAs using Lipofectamine^®^ 2000 Transfection Reagent (Invitrogen, Carlsbad, CA, USA) according to the manufacturer’s protocol. Forty-eight hours after transfection, the BMDCs were collected for analysis. We performed RT-PCR to determine their Jagged1 mRNA expression.

We co-cultured siRNA-modified BMDCs in 24-well plates with T cells stimulated with rLACK (100 µg/µl). The culture supernatants were collected 72 h later for cytokine analysis and the cells were collected for flow cytometry and qRT-PCR analysis.

### Flow Cytometric Analysis

For cell surface marker analysis, we stained cell samples with fluorochrome-conjugated mAbs (BD Biosciences, San Jose, CA, USA) for 30 min, then washed the samples three times with 2% FBS in PBS. For intracellular cytokine or transcription factor analysis, cells were stimulated with 50 ng/ml phorbol myristate acetate plus 500 ng/ml ionomycin for 4 h in the presence of 1 µg/ml brefeldin A (BD Biosciences, San Jose, CA, USA), then fixed and permeabilized, followed by cytoplasmic staining with the appropriate fluorochrome-conjugated mAbs (eBioscience, San Diego, CA, USA). We performed sample acquisition with a FACS Aria II (BD Biosciences, Franklin Lakes, NJ, USA) and analyzed the results using FlowJo software.

### Analysis of Macrophage and DCs Activation Induced by the LACK_156–173_ Epitope

#### Cell Viability Assay

RAW264.7 cells were purchased from the American Type Culture Collection (Manassas, VA, USA). The effect of rLACK on the viability of RAW264.7 cells was analyzed using the 3-(4,5-dimethylthiazol-2-yl)-2,5-diphenyltetrazolium bromide (MTT) assay. Cells were pre-incubated in 96-well plates for 24 h, then incubated with rLACK (100 µg/µl) or LPS (1 µg/ml) for 24 h. After incubation, MTT stock solution (2 mg/ml) was added to each well, and the cells were incubated for an additional 2 h. After incubation, the medium was removed and isopropyl alcohol was added to solubilize the formazan salt. The amount of formazan salt was determined by measuring the absorbance at 595 nm using an ELISA microplate reader (Model 550, Bio-Rad, Hercules, CA, USA).

#### Targeting Pattern Recognition Receptors (PRRs)

To investigate the role of PRRs in the macrophage activation induced by rLACK, RAW264.7 macrophages in 12-well plates were pre-incubated with medium containing the toll-like receptor (TLR) 2-specific mAb T2.5; the TLR4-specific mAb MTS510; the mannose receptor (MR)-specific mAb MR5D3; the dectin-1-specific mAb AF1756; the dectin-2-specific mAb D2.11E4; the major histocompatibility complex (MHC) class II-specific mAb MAB0874 (10 µg/ml); the mixed inhibitor of the NOD-like receptor (NLR) NOD-IN-1 (25 µM), which has balanced inhibitory activity on NOD1 and NOD2; or the NOD1 inhibitor nodinitib-1 (5 µM) (MCE, Monmouth Junction, NJ, USA) for 1 h. Next, the cells were treated with rLACK (100 µg/µl) or LPS (1 µg/ml) for 24 h. Then, we measured nitric oxide (NO) production, reactive oxygen species (ROS) generation, and cytokine production in the culture system.

To test the effects of rLACK on DCs deficient for PRRs directly, BMDCs obtained from BALB/c mice in 12-well plates were pre-incubated with medium containing the mAbs against PRRs for 1 h. Next, the cells were treated with rLACK (100 µg/µl) for 24 h. The mRNA expression of cytokines and surface molecules of BMDCs were measured by qRT-PCR.

To investigate if targeting PRRs influenced the effects of the LACK_156–173_ epitope in CAIA mice, we injected 100 µg of mAbs against PRRs i.p. on day 4. The mice were scored daily until day 14. The gene expression levels of inflammatory mediators in the knee joint were measured by qRT-PCR.

#### NO Assay

We assayed the supernatants from the RAW264.7 cell cultures for NO production. Each supernatant was mixed with an equal volume of Griess reagent (Sigma-Aldrich, St. Louis, MO, USA), then the light absorbance of the mixture at 540 nm was determined using a Multiskan™ Spectrum Microplate reader. The nitrite concentration was determined using a standard curve generated from dilutions of a standard nitrite solution.

#### Measurement of ROS Generation

We assessed the changes in fluorescence resulting from the oxidation of the fluorescent probe H2DCFDA (Invitrogen, Carlsbad, CA, USA) to evaluate the levels of intracellular ROS. RAW264.7 cells were incubated with 10 µM H2DCFDA for 1 h at 37°C. The degree of fluorescence was detected (485 nm, excitation; 535 nm, emission) using a microplate spectrofluorometer. For analysis of intracellular ROS production, images of the stained cells were captured using a fluorescence microscope (ECLIPSE 50, Nikon, Tokyo, Japan).

### Cytokine Production

The cytokine levels in the culture supernatants were evaluated with a Mouse Th17 Magnetic Bead Panel Kit (Merck Millipore, Burlington, MA, USA) according to the manufacturer’s instructions.

### Statistical Analysis

We used GraphPad Prism software (version 5.01) for the statistical analyses. We applied the Mann–Whitney *U* test or Student’s *t*-test for two-group comparisons. The data are displayed as the mean ± SEM. A *p*-value <0.05 was considered statistically significant.

## Results

### LACK_156–173_ Epitope Attenuates Arthritis Severity and Regulates Effector T Cell Responses

**Figure 1:**
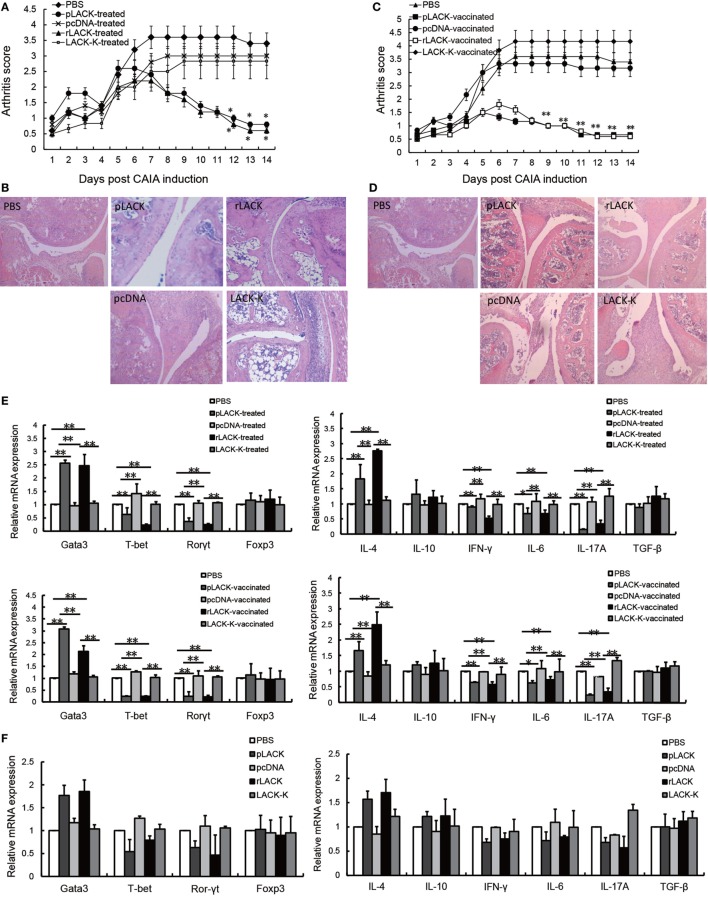

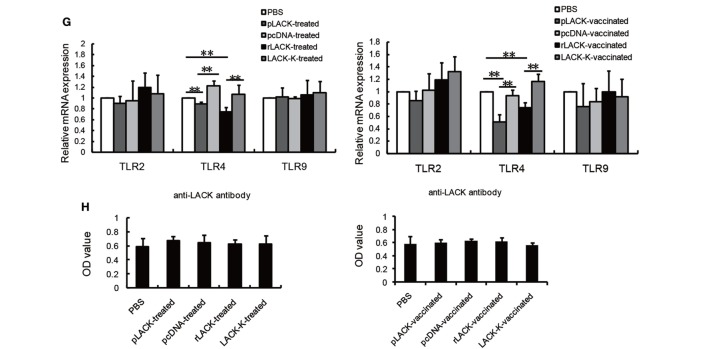
The effect of the LACK_156–173_ epitope in mice with collagen antibody-induced arthritis (CAIA). CAIA mice in the treatment groups were injected with PBS/pLACK/pcDNA (100 µg) or PBS/rLACK/LACK-K (50 µg) on day 4. CAIA mice were vaccinated 7 days before monoclonal antibody (mAb) injection. **(A–D)** The effects of Plack/rLACK treatment **(A,B)** and vaccination **(C,D)** on the arthritis scores and histopathology of CAIA mice. Original magnification, 100×. Eight animals in each group. **(E)** T cell transcription factor and cytokine gene expression in the knee synovium of CAIA mice at day 14. **(F)** The gene expression levels in normal mice injected with the LACK_156–173_ epitope. Normal mice were injected with PBS/pLACK/pcDNA (100 µg) or PBS/rLACK/LACK-K (50 µg). 7 days later, T cell transcription factor and cytokine gene expression in the knee joints of mice were determined by quantitative real-time polymerase chain reaction. **(G)** Toll-like receptors (TLRs) gene expression in the knee synovium of CAIA mice at day 14. **(H)** Anti-LACK_156–173_ epitope antibodies in the sera of CAIA mice were determined by enzyme-linked immunosorbent assay at day 14. The data are expressed as the mean ± SEM for five animals in each group, combined from at least three independent experiments. *p < 0.05, **p < 0.01.

The altered LACK-K peptide does not induce IL-4 production by Vβ4^+^/Vα8^+^ CD4^+^ T cells in BALB/c mice. The difference resulting from the single amino acid substitution suggests limited diversity in the endogenous T cell repertoire that recognizes LACK (16). Therefore, we used LACK-K as a negative control for rLACK in our research. To investigate the anti-inflammatory and immunomodulatory effects of the LACK_156–173_ epitope in arthritis, we synthesized rLACK (the synthetic polypeptide of the LACK_156–173_ epitope) and pLACK (the recombinant LACK_156–173_ epitope expression plasmid), and evaluated their effect on the development of CAIA, an animal model of RA. The clinical scores of the pLACK- and rLACK-treated or -vaccinated groups were significantly lower than those of the control arthritis groups (Figures 1A,C). Histological examination of the knees confirmed that the LACK_156–173_ epitope ameliorated arthritis, with regard to synovitis and the erosion of the cartilage and bone (Figures 1B,D).

To determine if the epitope affected cytokine production and T cell proliferation, we examined the gene expression levels of inflammatory mediators in the knee synovium. The gene expression levels of IL-4 and the Th2 cell transcription factor Gata3 were significantly higher in the pLACK- and rLACK-treated or -vaccinated groups than in the control arthritis group. Moreover, the gene expression levels of the pro-inflammatory cytokines IFN-γ, IL-6, and IL-17A, and the Th1 and Th17 cell transcription factors T-bet and Rorγt, were lower in the pLACK- or rLACK-injected mice (Figure 1E). We also examined the gene expression levels of the cytokines and effector T cell transcription factors in normal mice treated with pLACK or rLACK, which led to similar, but weaker, results (Figure 1F). These data indicate that both vaccination and treatment with the LACK_156–173_ epitope inhibit joint inflammation and modulate effector T cell response, by inducing Th2 cell responses and downregulating Th1 and Th17 cell responses in CAIA mice.

Toll-like receptors recognize microbial products and are involved in the regulation of T cell activation (21, 22), so we examined the gene expression levels of TLR2, 4, and 9 in the knee synovium. The TLR4 gene expression levels were significantly lower in the pLACK- and rLACK-treated or -vaccinated groups than in the controls (Figure 1G). These results show that the LACK_156–173_ epitope decreased the expression of TLR4 in CAIA mice, which may influence T cell activation.

The levels of anti-LACK_156–173_ epitope antibodies in the serum were not statistically different among the groups (Figure 1H), suggesting that pLACK and rLACK injection did not induce the production of antigen-specific antibodies. The effects of pLACK and rLACK in CIA mice were similar in above experiments. So we consider the polypeptide and the recombinant expression plasmid of the LACK_156–173_ epitope have equivalent potential for treatment.

### The Effect of the LACK_156–173_ Epitope Is Dependent on MHC Class II Molecule I-A^d^

**Figure 2:**
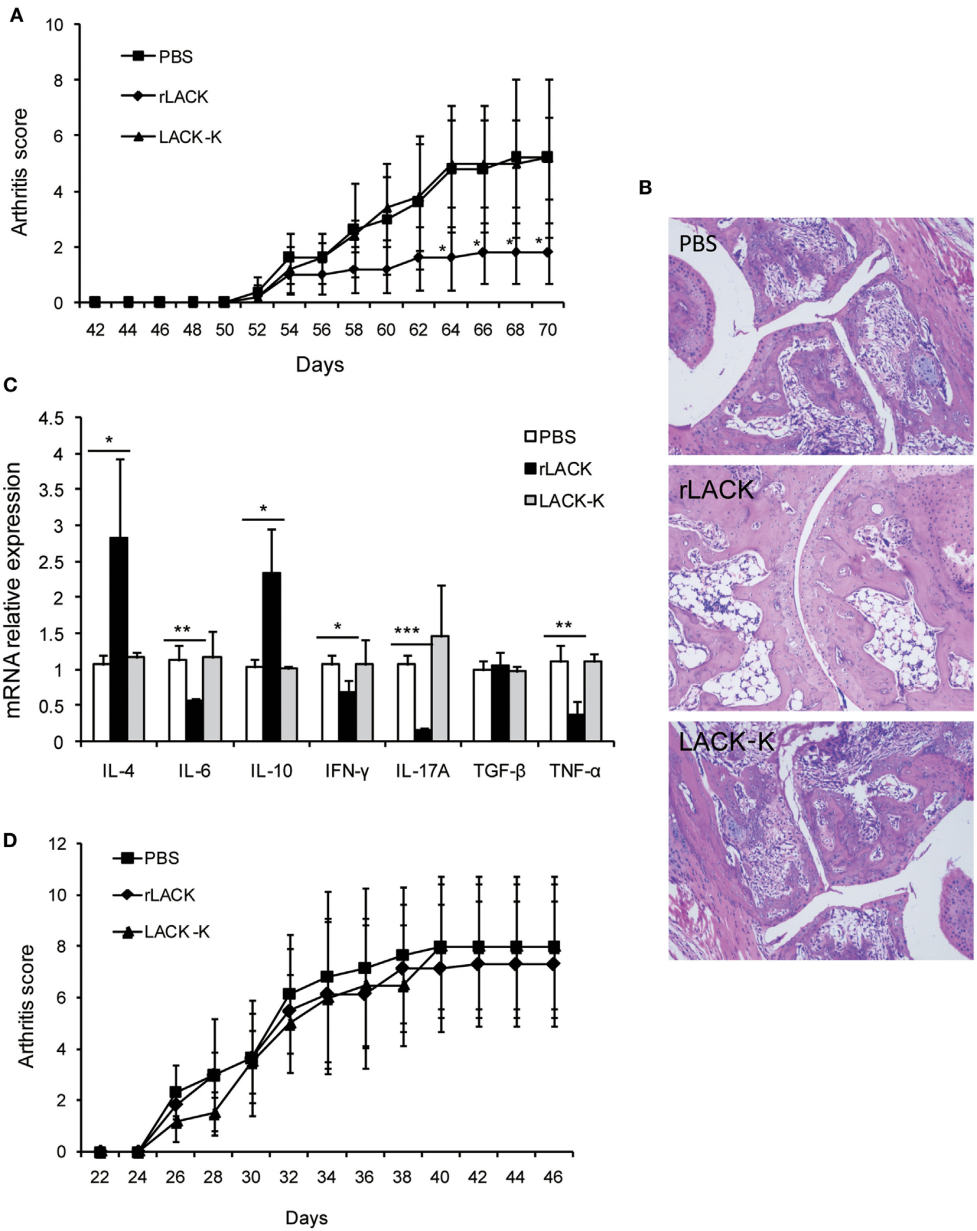
The effect of the LACK_156–173_ epitope in other animal models of autoimmune arthritis. Proteoglycan (PG)-induced arthritis (PGIA) mice were treated with PBS/rLACK/LACK-K (50 µg) after the third injection of PG with 7-day interval. Collagen-induced arthritis (CIA) mice were treated with PBS/rLACK/LACK-K (50 µg) after the booster injection with 7-day interval. **(A,B)** The severity of arthritis was evaluated by arthritis scoring **(A)** and joint histopathology **(B)** in PGIA mice. Original magnification, 100×. **(C)** Cytokine gene expression in the knee synovium of PGIA mice was analyzed on day 70. **(D)** The effect of LACK_156–173_ epitope treatment on the arthritis scores of CIA mice. The data are expressed as the mean ± SEM for eight animals in each group, combined from at least three independent experiments. *p < 0.05, **p < 0.01, ***p < 0.001.

The LACK_156–173_ epitope is presented by MHC class II I-A^d^ molecules, and recognized by Vβ4^+^/Vα8^+^ T cells. To further investigate the therapeutic potential of the LACK_156–173_ epitope, we administered it to PGIA and CIA mice, which are murine models of autoimmune arthritis. CIA is induced in DBA/1 mice (MHC class II I-A^q^), which have different MHC molecules with BALB/c mice (MHC class II I-A^d^). We found that rLACK peptide treatment led to significantly lower clinical scores and fewer histopathological alterations in PGIA mice than those of the arthritis controls (Figures 2A,B). In the rLACK-treated group, IFN-γ, TNF-α, IL-6, and IL-17A were markedly reduced to the levels found in the control mice. Meanwhile, rLACK treatment increased IL-4 and IL-10 levels in the knee synovium (Figure 2C). Thus, treatment with rLACK has therapeutic effects in PGIA mice. However, we did not observe similar results in CIA mice (Figure 2D), perhaps due to the lack of MHC class II I-A^d^ molecules in DBA/1 mice.

### The LACK_156–173_ Epitope Modulates the Differentiation and Maturation of DCs in CAIA Mice, Thereby Influencing T Cell Polarization

As antigen-presenting cells (APCs), such as DCs, are key players during both the initiation and progression of autoimmune responses, which include effector T cell responses (23, 24). To further explore the modulation of the adaptive immune response by the LACK_156–173_ epitope, we measured the quantity of CD11c^+^ DCs in the spleen, as well as their surface marker and cellular signaling pathway molecule expression.

**Figure 3:**
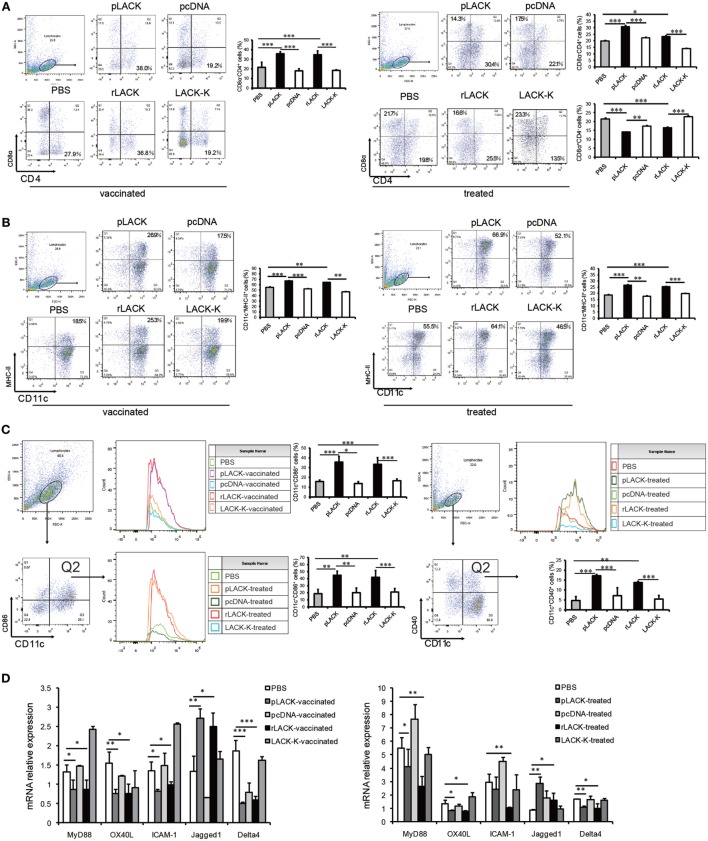
The effects of LACK_156–173_ on the subsets of CD11c^+^ dendritic cells (DCs), and their maturity and signaling pathways, in collagen antibody-induced arthritis (CAIA) mice. Single-lymphocyte suspensions from the spleens of CAIA mice were generated by mechanical disruption and red blood cell lysis 7 days after monoclonal antibody (mAb) injection. CD11c^+^ DCs were purified from splenocytes by magnetic cell sorting. **(A)** The levels of the surface molecules CD8α and CD4 on splenic DCs from LACK_156–173_ epitope-vaccinated or -treated CAIA mice 7 days after injection of the mAb were quantified by flow cytometry. **(B)** The levels of the surface molecules CD11c and MHC class II on splenic DCs from LACK_156–173_ epitope-vaccinated or -treated CAIA mice 7 days after injection of the mAb were quantified by flow cytometry. **(C)** The levels of the surface molecules CD11c, CD86, and CD40 on splenic DCs from LACK_156–173_ epitope-vaccinated or -treated CAIA mice 7 days after injection of the mAb were quantified on FACS by double staining. **(D)** Co-stimulatory molecule, adhesion molecule, toll-like receptor (TLR)4 signaling pathway molecule, and Notch ligand gene expression in DCs from CAIA mice 7 days after the injection of the mAb. One representative of three independent experiments is shown. The results are expressed as the mean ± SEM. *p < 0.05, **p < 0.01, ***p < 0.001.

We found significantly more CD4^+^CD8α^−^CD11c^+^ DCs in the pLACK- and rLACK-vaccinated CAIA mice than in the controls (Figure 3A), indicating that the epitope affected DC subsets (25, 26). We found a greater number of DCs expressing the surface molecules MHC class II and CD86 in the pLACK- or rLACK-vaccinated CAIA mice (Figures 3B,C), suggesting that the epitope promoted DC maturation (27, 28). The numbers of CD4^+^CD8α^−^CD11c^+^ DCs were also significantly higher and the numbers of CD4^−^CD8α^+^CD11c^+^ DCs were lower in the pLACK- and rLACK-treated groups than in the controls. We observed a higher proportion of DCs expressing the surface molecules MHC class II, CD40, and CD86 in the pLACK- and rLACK-treated mice than in the controls (Figures 3A–C). The gene expression levels of the pro-inflammatory co-stimulatory molecule OX40L, the adhesion molecule ICAM-1, and the TLR4 signaling pathway molecule MyD88 were significantly lower in the pLACK- and rLACK-treated and -vaccinated groups. The gene expression of the Notch ligand Delta4, which has been reported to direct the differentiation of Th17 cells (29, 30), was significantly lower in the pLACK- and rLACK-treated or -vaccinated groups. On the contrary, gene expression of the Notch1 ligand Jagged1 was significantly higher than in the controls, which confirmed that the epitope drives the differentiation of Th2 cells (Figure 3D). Taken together, these results demonstrate that CD11c^+^ DC subsets, maturity, intracellular signaling pathways, and interactions with T cells are modulated by the LACK_156–173_ epitope.

**Figure 4:**
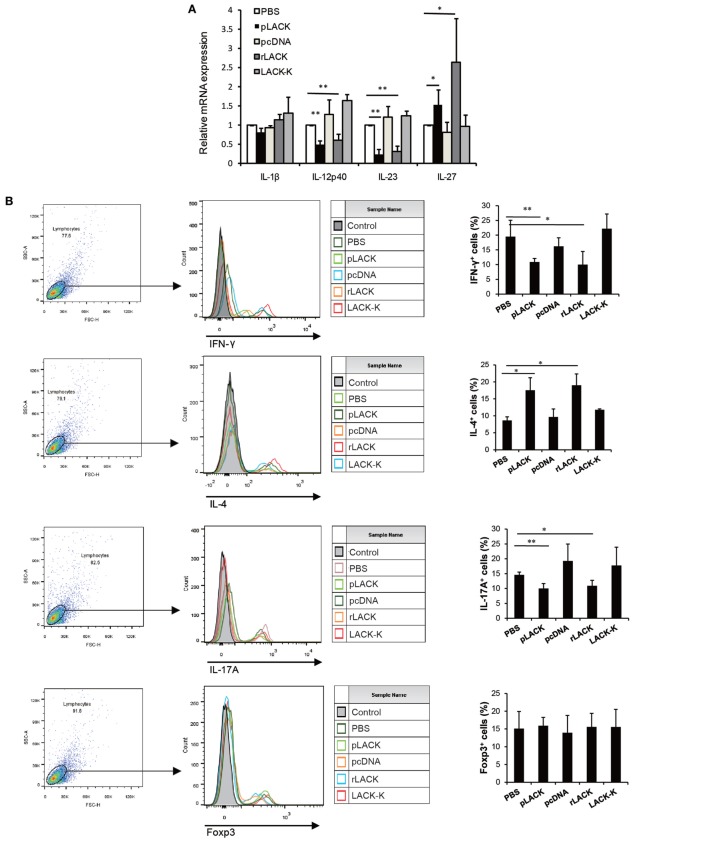

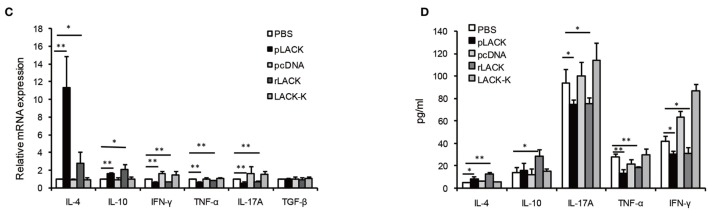
The effect of LACK_156–173_ epitope-matured dendritic cells (DCs) in collagen antibody-induced arthritis (CAIA) mice on T cell polarization and function *in vitro*. LACK_156–173_ epitope-matured CD11c^+^ DCs were prepared from the spleens of CAIA mice, as described in Section “Analysis of the Phenotypes of DCs and the *In Vitro* Polarization of T Cells From CAIA Mice.” CD11c^+^ DCs were co-cultured for 72 h with T cells, isolated *via* nylon wool fiber in a syringe, from the spleens of BALB/c mice that had been i.p. injected with 100 µg OVA_323–339_ 7 days before isolation. **(A,C)** Cytokine mRNA expression. **(B)** The numbers of interleukin (IL)-4^+^ cells, IFN-γ^+^ cells, IL-17A^+^ cells, and Foxp3^+^ cells were measured by flow cytometry. **(D)** The concentrations of the cytokines in the culture supernatants. One representative of three independent experiments is shown. Results are expressed as mean ± SEM. *p < 0.05, **p < 0.01.

To further investigate the functions of DCs that are affected by the LACK_156–173_ epitope, we co-cultured DCs from CAIA mice treated with the LACK_156–173_ epitope with T cells *in vitro*. The gene expression levels of the pro-inflammatory cytokines IL-12p40 and IL-23 were significantly lower and the level of the anti-inflammatory cytokine IL-27 was higher in the cultures with the cells from the pLACK- and rLACK-treated groups (Figure 4A), indicating that the LACK_156–173_ epitope regulates cytokine secretion by DCs. The numbers of IFN-γ^+^ cells and IL-17A^+^ cells were significantly lower, and the number of IL-4^+^ cells was higher, in the cultures with the cells from the pLACK- and rLACK-treated groups (Figure 4B). On the other hand, the cultures of the DCs from the LACK_156–173_ epitope-stimulated groups co-cultured with T cells had significantly lower IFN-γ, TNF-α, and IL-17A gene expression and secretion, and increased IL-4 and IL-10 production (Figures 4C,D), in comparison with the cultures containing non-epitope-stimulated DCs. These findings further support the idea that the LACK_156–173_ epitope polarizes DCs toward a Th2-promoting profile.

### The LACK_156–173_ Epitope Polarizes DCs and Induces Th2 Responses *via* Jagged1 Signaling

**Figure 5:**
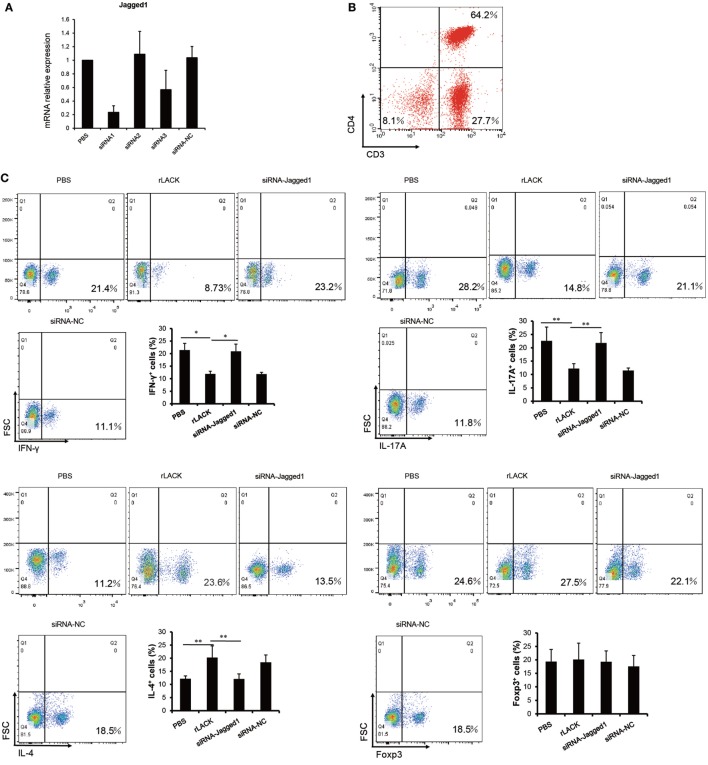

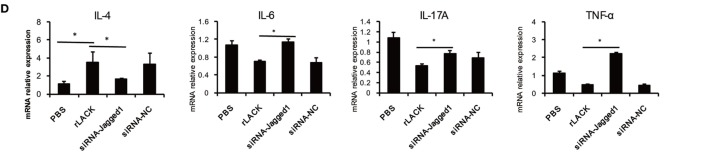
The effect of targeting Jagged1 signaling on the induction of T cell responses by LACK_156–173_ epitope-stimulated dendritic cells (DCs). siRNA-modified bone marrow-derived dendritic cells (BMDCs) were co-cultured with T cells stimulated by rLACK (100 µg/µl). **(A)** Three siRNAs targeting different regions of Jagged1 mRNA were designed and purchased together with a scrambled siRNA to serve as a negative control. Harvested BMDCs were transfected by using Lipofectamine with three siRNAs targeting different regions of Jagged1 mRNA and a scrambled siRNA to serve as a negative control. 24 h later, we performed quantitative real-time polymerase chain reaction (qRT-PCR) to determine their Jagged1 mRNA expression in the BMDCs. **(B)** The purity of CD3^+^CD4^+^ cells in isolated T cells was measured by flow cytometry. **(C)** The numbers of interleukin (IL)-4^+^ cells, IFN-γ^+^ cells, IL-17A^+^ cells and Foxp3^+^ cells in the co-culture system were examined using flow cytometry. **(D)** Cytokine mRNA expression was measured by qRT-PCR analysis. Dot plots are representative of one of three experiments. The results are expressed as the mean ± SEM, combined from at least three independent experiments.*p < 0.05, **p < 0.01.

Targeting Notch1 signaling on DCs has been proposed as a novel strategy for modulating Th2 immune responses (31). So, we evaluated if Notch1 signaling was necessary for the LACK_156–173_ epitope to polarize DCs, by using siRNA to silence Jagged1 expression in BMDCs, which were cultured from bone marrow cells of BALB/c mice. Forty-eight hours after transfection, we selected one of three siRNAs based on the levels of Jagged1 gene expression in the transfected cells (Figure 5A), then we co-cultured the siRNA-modified BMDCs with T cells. Also, we examined the purity of CD3^+^CD4^+^ cells in isolated T cells (Figure 5B). In the co-colure system, we found that the Jagged1 siRNA significantly reduced the number of IL-4^+^ cells induced by rLACK, whereas it increased the number of IFN-γ^+^ cells and IL-17A^+^ cells (Figure 5C). IL-4 production was also decreased, and IL-6, TNF-α, and IL-17A were upregulated by Jagged1 siRNA transfection (Figure 5D). Thus, the LACK_156–173_ epitope polarizes DCs to induce Th2 responses, mainly *via* Jagged1 signaling.

### Anti-MR Inhibits Macrophage Activation and the Functions of DCs Induced by the LACK_156–173_ Epitope

**Figure 6:**
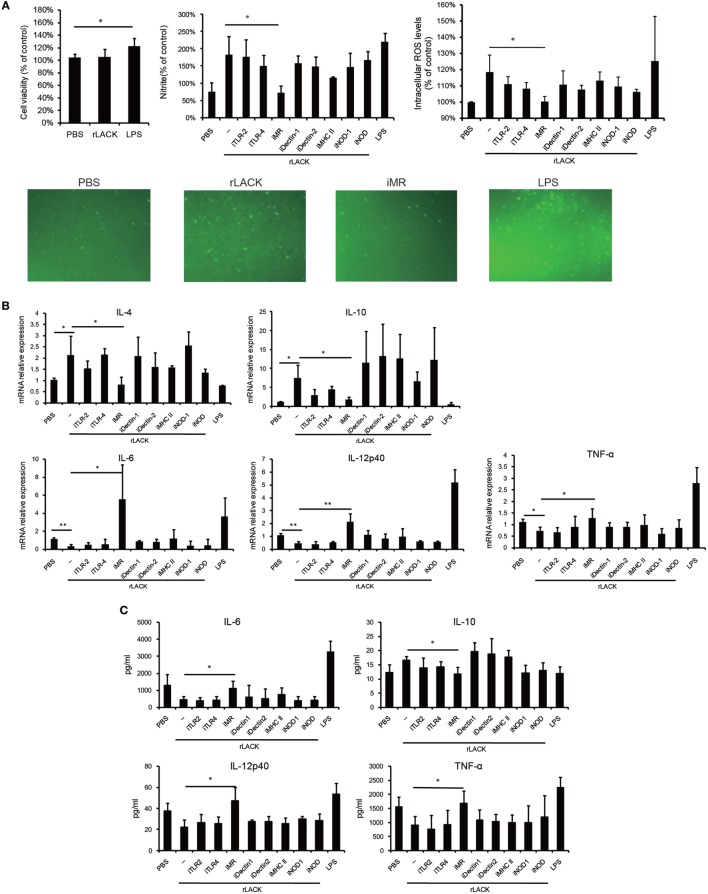
The effect of anti-mannose receptor (MR) on macrophage and dendritic cells (DCs) activation induced by the LACK_156–173_ epitope. RAW264.7 macrophages were pre-incubated with medium containing monoclonal antibodies (mAbs) against toll-like receptor (TLR)2, TLR4, MR, dectin-1, dectin-2, or MHC class II (10 µg/ml), or NOD-IN-1 (25 µM) or nodinitib-1 (5 µM) for 1 h. Then, the cells were treated with rLACK (100 µg/µl) or lipopolysaccharide (LPS) (1 µg/ml) for 24 h. **(A)** The effect of rLACK on cell viability was analyzed using an MTT assay. The supernatants of the RAW264.7 cell cultures were examined for NO production using Griess reagent. The degree of intracellular reactive oxygen species (ROS) generation was detected using a microplate spectrofluorometer. Images of the stained cells were captured by immunofluorescence microscopy. **(B)** Cytokine mRNA expression was measured by quantitative real-time polymerase chain reaction (qRT-PCR) analysis. **(C)** The concentrations of the cytokines in the culture supernatants. Data are expressed as the mean ± SEM combined from at least three independent experiments. *p < 0.05, **p < 0.01.

Innate immunity responds immediately to infection and damage; it is activated through the binding of conserved pathogen- or damage-associated molecules to PRRs on DCs and other innate immune cell types, and it directs the adaptive immune response (32). Most PRRs are expressed on both DCs and macrophages. Thus, we used RAW264.7 macrophages to investigate if the LACK_156–173_ epitope activates DCs through PRRs. We found that the rLACK peptide did not affect cell viability (Figure 6A). To investigate if rLACK could induce macrophage activation, we incubated RAW264.7 cells with rLACK or LPS for 24 h. We observed significantly higher NO and ROS generation in the cells treated with rLACK or LPS than in the controls (Figure 6A). IL-4 and IL-10 production were also increased, and IL-6, TNF-α, and IL-12p40 were downregulated, compared to their expression in the PBS control, by rLACK treatment (Figures 6B,C). These effects were abolished by using an anti-MR mAb, but not by targeting many other PRRs (Figures 6A–C). These data suggest that rLACK may bind MR to induce macrophage activation.

**Figure 7:**
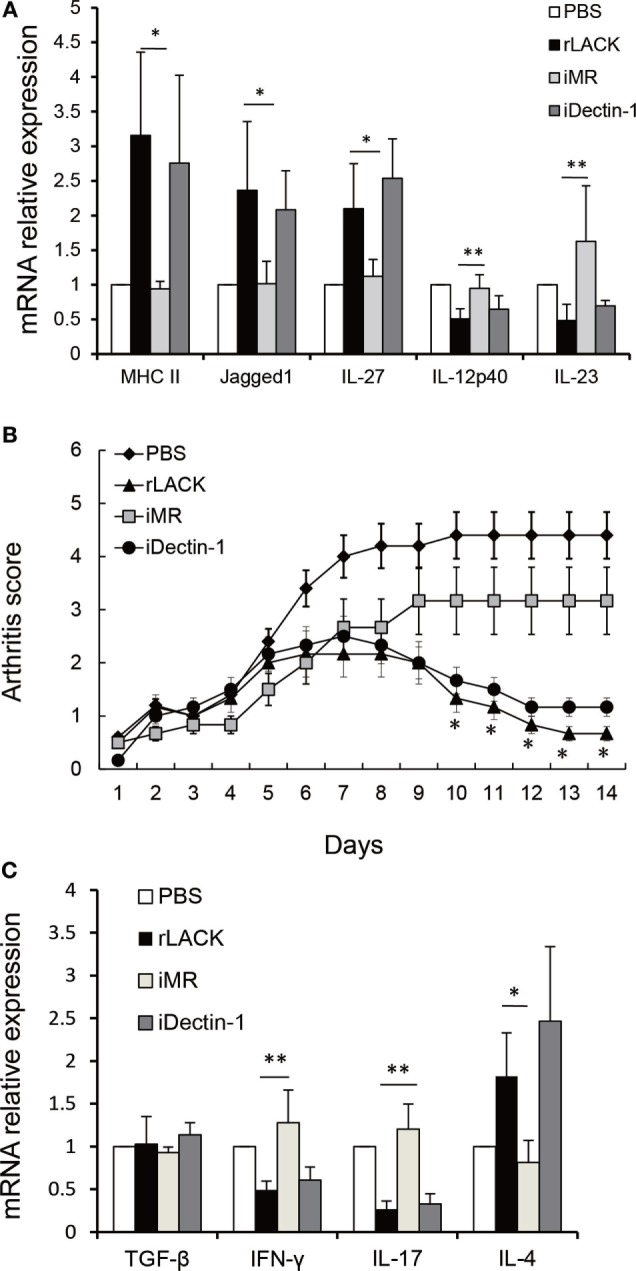
The effect of anti-mannose receptor (MR) on LACK_156–173_ epitope-stimulated dendritic cells (DCs) and rLACK-treated collagen antibody-induced arthritis (CAIA) mice. **(A)** Bone marrow-derived dendritic cells (BMDCs) obtained from BALB/c mice in 12-well plates were pre-incubated with medium containing the monoclonal antibodies (mAbs) against MR, dectin-1 (10 µg/ml) for 1 h. Next, the cells were treated with rLACK (100 µg/µl) for 24 h. The mRNA expression of cytokines and surface molecules of BMDCs were measured by quantitative real-time polymerase chain reaction. **(B)** The effect of targeting MR on the arthritis scores of CAIA mice treated with rLACK. The mAb against MR/Dectin-1 (100 µg) was injected i.p. on day 4. Eight animals in each group. **(C)** Cytokines gene expression in the knee synovium of CAIA mice at day 14. Data are expressed as the mean ± SEM for five animals in each group, combined from at least three independent experiments. *p < 0.05, **p < 0.01.

To further verify if rLACK activates DCs and modulates the functions of DCs through MR, we used BMDCs obtained from BALB/c mice, which were pre-incubated with mAb against MR for 1 h. The mAb against Dectin-1 was served as a control mAb. Then, the cells were treated with rLACK (100 µg/µl) for 24 h. We found the effect of upregulating MHC II and Jagged1 mRNA expression of BMDCs were abolished by anti-MR mAb, which indicated that LACK_156–173_ epitope induces DCs activation and Jagged1 signaling by binding MR. The effect of upregulating IL-27 mRNA expression of BMDCs, and downregulating IL-12p40, IL-23 mRNA expression by rLACK were also abolished by anti-MR mAb (Figure 7A), which indicated that LACK_156–173_ epitope modulates cytokines secretion of DCs by binding MR. Taken together, these results demonstrate that DC maturity and the modulatory effect on T cells of DCs are induced by the LACK_156–173_ epitope through MR. In addition, the therapeutic effect of rLACK in CAIA mice was blocked by anti-MR mAb injection (Figure 7B). The effect of upregulating IL-4 mRNA expression and downregulating IL-17, IFN-γ mRNA expression in knee joint by rLACK was also blocked by anti-MR mAb injection (Figure 7C). Therefore, we consider that the LACK_156–173_ epitope polarizes DCs, modulates effector T cell response, and induces remission of arthritis by binding MR.

## Discussion

Infectious microorganisms can exert immunoregulatory and immunosuppressive functions (33). Ova, extracts, and macromolecular substances are the most frequently used microorganism-derived immune-modulatory agents (34–36); they may induce infection or activate existing immunity. In this research, we first explored a single epitope of microorganism therapy with LACK_156–173_. We found that the epitope did not elicit inflammation and markedly ameliorated murine autoimmune arthritis. The therapeutic effects appeared to be mediated by the regulation of the differentiation, maturation, and function of DCs *via* MR, resulting in the upregulation of Jagged1 and leading to Th2 polarization.

The single epitope LACK_156–173_ could drive naive T cells into a Th2 response in BALB/c mice. In our research, it does not only induce Th2 inflammatory response but also inhibit the Th1 and Th17 pathways, and blocked the production of pro-inflammatory cytokines. Due to the pathologic role of pro-inflammatory cytokines, such as TNF-α, in RA, cytokine inhibitors have been developed for the treatment of RA. However, these biological drugs are effective in a limited number of patients. Our research demonstrated that the LACK_156–173_ epitope, which has broad regulatory effects on the balance of T cell subsets, may induce a better response in a wider variety of RA patients. H164, a positively charged amino acid within the LACK_156–173_ epitope (ICFSPSLEHPIVVSGSWD), determines its binding affinity for MHC class II molecules and the negatively charged motif in the T cell receptor (TCR) β chain (16). Therefore, LACK-K164 did not elicit responses in BALB/c mice. Consequently, H164 in the LACK_156–173_ epitope seems to be very important for the therapeutic effect of the epitope.

Dendritic cells, the most powerful APCs, carry antigens to the draining lymph nodes, where they promote the activation, differentiation, and polarization of naive T cells into effector T cell subsets. Conventional DCs (cDCs) are professional APCs that specialize in these functions. After contact with antigens, DCs undergo a process of maturation, then migrate to the T cell areas of lymph nodes. The maturation process includes the increased expression of MHC and co-stimulatory molecules, such as CD40, CD80, CD86, and CD54; decreased antigen capture and phagocytic capacity; enhanced cytokine secretion; and different patterns of chemokine receptor expression and chemokine production, enabling DC migration and the recruitment of other cell types (37, 38). CD8α^−^ cDCs are the most efficient at Th2 cell polarization, whereas CD8α^+^ cDCs are particularly efficient at Th1 cell activation (19, 39, 40). Our data showed that LACK_156–173_ epitope injection resulted in the expansion of CD4^+^CD8α^−^CD11c^+^ DCs; a reduction in CD4^−^CD8α^+^CD11c^+^ DCs; increased expression of MHC class II, CD40, and CD86; and decreased pro-inflammatory cytokine, co-stimulatory molecule (OX40L), adhesion molecule (ICAM-1), and TLR4 signaling pathway molecule expression among DCs. Based on our findings, we speculate that the differentiation, maturation, and functions of CD11c^+^ DCs are modulated by the LACK_156–173_ epitope. In addition, LACK_156–173_ epitope-matured DCs promoted Th2 responses and inhibited Th1 and Th17 responses, both *in vivo* and *in vitro, via* Jagged1. Therefore, we believe that the LACK_156–173_ epitope polarizes DCs and induces Th2 responses, mainly *via* Jagged1 signaling.

T cell activation requires recognition of an antigen that has been processed into a peptide, then loaded into a specialized groove in an MHC class II molecule for presentation to a TCR. The cells capable of processing and presenting antigen in this way include DCs, macrophages, and B cells. The LACK_156–173_ epitope is presented by MHC class II I-A^d^ molecules, which are recognized by Vβ4^+^/Vα8^+^ TCRs. So, the LACK_156–173_ epitope has therapeutic effects on autoimmune arthritis in BALB/c mice (I-A^d^), but not DBA/1 (I-A^q^) mice. Meanwhile, H164 in the LACK_156–173_ epitope seems to be important for the therapeutic effect. So, changes in this amino acid or modification of the LACK_156–173_ epitope make it can be identified by other MHC molecules and act on other organisms are our future research direction.

**Figure 8:**
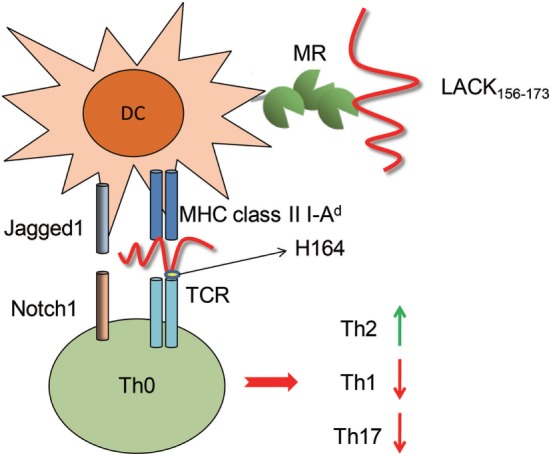
The effect of the LACK_156–173_ epitope on T cell polarization. The LACK_156–173_ epitope regulates the subsets, maturation, and functions of dendritic cells through binding to mannose receptor (MR), resulting in upregulation of Jagged1. The LACK_156–173_ epitope is presented by major histocompatibility complex (MHC) class II I-A^d^ molecules to Vβ4^+^/Vα8^+^ T cells and induces T helper (Th)2 cell polarization. Its effects are dependent on residue H164.

In another hand, we did not observe an effect of blocking MHC class II molecules on LACK_156–173_ epitope-induced cell activation. This phenomenon suggests that the MHC class II molecules are responsible only for antigen presentation, not for the capture of the LACK_156–173_ epitope. In addition, our results indicate that MR on DCs binds to rLACK and induces cell polarization. PRRs include TLRs, NLRs, RIG-I-like receptors, and C-type lectin receptors (CLRs). PRRs play important roles in determining the class of the adaptive immune response to a stimulus. MR (CD206), a member of the C-type lectin family, is highly expressed on macrophages and DCs (41). DCs drive Th2 differentiation *via* the activation of a combination of CLRs, including MR (42). MR does not appear to function as a canonical PRR capable of independent signal transduction, due to the lack of signaling motifs in its cytoplasmic tail. Signal transduction through MR has not been well studied. However, we confirmed that the LACK_156–173_ epitope regulates DCs by binding MR, through which it modulates T cell responses (Figure 8).

In summary, we confirmed that single microorganism epitope therapy with LACK_156–173_ did not cause inflammation and markedly ameliorated murine autoimmune arthritis. Our results demonstrate the dramatic therapeutic potential of the LACK_156–173_ epitope in autoimmune arthritis.

## Ethics Statement

All animal experiments were carried out and approved according to the guidelines of the animal care and use committee at Harbin Medical University.

## Author Contributions

FY and YL conceived and designed the experiments. FY, XF, and HH performed all experiments and analyzed the data. FY, XF, HH, QD, HL, and YL wrote the paper and edited the manuscript. All authors read and approved the final manuscript.

## Conflict of Interest Statement

The authors declare that the research was conducted in the absence of any commercial or financial relationships that could be construed as a potential conflict of interest.
